# Food safety practice and associated factors in public food establishments of Ethiopia: A systematic review and meta-analysis

**DOI:** 10.1371/journal.pone.0268918

**Published:** 2022-05-27

**Authors:** Aiggan Tamene, Aklilu Habte, Demelash Woldeyohannes, Abel Afework, Fitsum Endale, Addisalem Gizachew, Dawit Sulamo, Legesse Tesfaye, Mihretu Tagesse

**Affiliations:** 1 School of Public Health, College of Medicine and Health Sciences, Wachemo University, Hosaena, Ethiopia; 2 Dilla University Comprehensive Referral Hospital, Dilla University, Dilla, Ethiopia; The Technical University of Kenya, KENYA

## Abstract

**Background:**

In terms of global impact, foodborne infections have been likened to major infectious diseases such as HIV/AIDS, malaria, and tuberculosis, with 1 in 10 people becoming ill and 420,000 deaths per year. A large number of these incidents are caused by improperly handled food in foodservice establishments. Therefore, this systematic review and meta-analysis aims to estimate the proportion of food handlers in Ethiopian commercial food service establishments who have safe food handling practices and their associated factors.

**Methods:**

Studies conducted before 02-05-2022 were explored in PubMed, Science Direct, Web of Science, Scopus, Embase, Google Scholar, ProQuest, and Ovid MEDLINE^®^, as well as other sources. A total of fourteen studies were included in the final synthesis. Data were extracted using a standardized data extraction format prepared in Microsoft excel and the analysis was done using STATA 16 statistical software. The quality of included studies was assessed using the Joanna Briggs Institute’s Critical assessment checklist for prevalence studies. To evaluate publication bias, a funnel plot and Egger’s regression test were employed. The I^2^ statistic was calculated to examine for study heterogeneity. To assess the pooled effect size, odds ratios, and 95% confidence intervals across studies, the DerSimonian and Laird random-effects model was used. Subgroup analysis was conducted by region and publication year. The influence of a single study on the whole estimate was determined via sensitivity analysis.

**Results:**

Of 323 identified articles, 14 studies were eligible for analysis (n = 4849 participants). The pooled prevalence estimate of safe food handling practices among Ethiopian food handlers was 47.14% (95% CI: 39.01–55.26, I^2^ = 97.23%). Foodservice training (OR, 3.89; 95% CI: 2.37–5.40), having on-site water storage facilities (OR, 4.65; 95% CI: 2.35–6.95), attitude (OR, 4.89; 95% CI: 1.39–8.29), hygiene and sanitary inspection certification (OR, 3.08; 95% CI: 1.62–4.45) were significantly associated with safe food handling practice among food handlers.

**Conclusion:**

This review identified that improvements are needed in food handling training, government regulation, and infrastructure. Standard regulations for food service enterprises must be implemented on a local and national level. Though long-term food safety requires legislation and training, failure to address infrastructure challenges can harm public health efforts. Access to safe drinking water and the presence of sanitary waste management systems should all be part of the basic infrastructure for ensuring the safety of food in food businesses.

## Introduction

Food safety is defined as the conditions and measures that must be in place during the production, processing, storage, distribution, and preparation of food to ensure that it is safe, sound, wholesome, and fit for human consumption [1]. When people have access to safe food, their health improves. Food safety enhances health and productivity while also providing a firm platform for development and poverty reduction [2].

Foodborne diseases (FBDs) are a broad category of illnesses caused by microbial infections, parasites, chemical pollutants, and bio-toxins in food. Unsafe food causes more than 200 diseases—ranging from diarrhea to cancers [3]. The global burden of foodborne diseases has been reported to be comparable to major infectious diseases such as HIV/AIDS, malaria, and tuberculosis, with 1 in 10 people becoming ill and 420,000 deaths annually [4]. 110 billion U.S dollars are lost each year in medical expenses resulting from unsafe foods in low- and middle-income countries (LMICs). In regional terms, African figures suggest that each year, about 91 million people become ill and 137,000 people die due to foodborne diseases [5].

A significant number of foodborne disease cases are caused by meals that have been incorrectly prepared or handled in food service establishments. Not all food handlers understand the roles they must play to protect their health and that of the wider community [6]. The number of individuals buying and eating food produced in public venues has increased as a result of urbanization and changes in consumer behavior. Food handlers now have a greater obligation to ensure food safety as a result of these changes [7]. Accordingly, food safety is crucial to protect consumers from health risks related to foodborne illnesses. In and of itself, this is a good enough goal to reach using proper handling procedures, but there are other reasons, too. Safe food products shield businesses and stakeholders from costly penalties and legal action. Fines and legal consequences could close down a facility and even bankrupt an establishment [8].

While protocols in the preparation, handling and storage of food may vary depending on the food prepared and the establishment in which it is served, the World Health Organization (WHO) advises "five keys to food safety" to prevent foodborne infections. These five simple keys to safe and healthy food are: keep clean, separate raw and cooked foods, cook thoroughly, keep food at safe temperatures, and use safe water and raw materials [9]. Any deviation from these recommendations in a business with hundreds or thousands of consumers has the potential to affect a large number of people.

Ethiopia’s food systems are already under strain due to population increase, urbanization, and a lack of resources [10]. According to numerous studies undertaken in Ethiopia, safe food handling practices in food establishments range from 20% to 70%. Food handler hygiene, food safety training, facility sanitary conditions, the lack of disposal services, the legal status of the license, and environmental hygiene were all highlighted as key drivers of safe food handling [11–15].

Considering the amount of food safety studies, Ethiopian researchers have yet to investigate the possible causes of variability and inconsistency in the commercial sector’s embrace of safe food handling practices. The limited literature on Ethiopian food safety regulation reveals that we still lack viable models for standards and techniques that can work at scale to ensure food safety in situations where risks are prevalent, compliance costs are high, and enforcement capability is poor.

Food safety standards or legislation governing the preparation, composition, and marketing of food intended for human consumption should be based on all available scientific information and data to attain a high level of protection for human health and life. The ideas that have worked in developed countries cannot be automatically adapted to developing countries due to the vast differences in food systems and regulatory settings [16]. It is therefore vital to employ systematic reviews and meta-analyses to identify, select, and critically appraise relevant evidence about safe food handling practices and their associated factors among food handlers in Ethiopian commercial foodservice facilities. The findings will help to consolidate earlier findings and demonstrate the effects of relevant variables in safe food handling practice. Furthermore, identifying the antecedents of safe food handling that have the most significant effects vs those that have smaller effects might aid scholars, practitioners, and policymakers in determining a course of action.

## Methods and materials

### Review typology

A systematic review was done to appraise and synthesize existing evidence, identify research gaps in the evidence base, and make recommendations. For this review and meta-analysis, the Preferred Reporting Items for Systematic Reviews and Meta-Analysis guideline (PRISMA) was used (S1 File).

### Information sources and search strategies

The following databases: PubMed/MEDLINE, Google Scholar, African Journal Online (AJOL), Hinari, Science Direct, ProQuest, Directory of Open Access Journals, POPLINE, Ovid MEDLINE, and Cochrane Library were searched from inception to 2022-03-05. The search syntax was created for PubMed initially, and subsequently modified to meet the additional database-specific search requirements. The following key terms were used in combination with Boolean operators “AND” and “OR”: ((("Food Safety"[MeSH Terms] OR "Food hygiene"[All Fields] OR "Food sanitation"[All Fields] OR "Food handling"[All Fields]) AND "Practice"[All Fields] AND "Associated factors"[All Fields]) OR "Related factors"[All Fields] OR "Determining factors"[All Fields]) AND ("Food handler"[All Fields] OR "Food handlers"[All Fields]) AND ("Ethiopia"[MeSH Terms] OR "Ethiopia"[All Fields] OR "Ethiopia’s"[All Fields]) (S2 File).

The electronic database search was then supplemented with grey literature identified from Google scholar, Google search, and Ethiopian University digital repositories (such as the Addis Ababa University Digital Library, and Jimma University Digital Library). Reference lists of included studies were also scanned to ensure a complete search of the literature.

### Eligibility criteria

#### Inclusion criteria

*Study designs*. All types of observational studies.

*Setting*. All papers reporting on food safety practice, and associated factors among Ethiopian food handlers were included in this systematic review and meta-analysis regardless of their study area.

*Time frame*. There was no restriction on the study date. All studies reported up to Feb 05, 2022, were considered.

*Publication condition*. This review included articles published in peer-reviewed journals and relevant grey literature.

*Language*. only articles reported in English were considered.

#### Exclusion criteria

Qualitative studies, reviews, commentaries, letters to editors, interventional studies, as well as other opinion publications were excluded from the analysis. Before being included in the final review and meta-analysis, the title, abstract, and full text of articles were analyzed and assessed. As assessing methodological quality in the absence of the complete text was difficult, studies that were not accessed after at least two email contacts with the primary authors were removed. Studies that appeared under multiple search terms were published in a language other than English, self-identified as pilot/feasibility work, follow-up work with no new outcome measures, and studies with multiple (salami) publications were all excluded.

### Screening

Endnote X9.3.3 (Thompson Reuter, CA, USA) was used to import and de-duplicate all of the references. AT and AA screened all references at the title, abstract, and full-text stage, with 20% of them being screened again. At each stage, the criteria for exclusion were recorded. A reference was included in the next round of reviewing in case there was any doubt. A third reviewer (DW) independently screened 10% of the titles, abstracts, and full texts that were removed; the investigators collated the screened articles, and any differences were resolved by consensus.

### Study selection and data extraction

AT and AA used an excel spreadsheet to extract descriptive data, such as the first author, type of publication, year of study/publishing, objectives, study region, study population (age, sample size, gender); study design, sample size, response rate; the proportion of safe food handling practice, odds ratio, and 95% confidence intervals (CIs).

### Outcome measurement

This study’s primary outcome was safe food handling practice. It was deemed a safe practice if/when food handlers followed recommended protocols during the storage, transportation, preparation, and serving of food to guarantee that it was safe, sound, wholesome, and fit for human consumption. We included research that met the criteria outlined above. Each included study’s operational definition was double-checked and described in a table (S4 File).

## Operational definition

### Food handler

A food handler is someone who works in a public food and beverage establishment and has direct contact with food or food utensils. This includes chefs and food preparation assistants (C&FPA), front of house staff (FOH), e.g. wait staff and bar staff, back of house staff (BOH), e.g. staff working in a non-customer facing role, often responsible for washing, cleaning, and sanitation such as kitchen porters

### Quality appraisal

The Joanna Briggs Institute (JBI) quality assessment tool for prevalence studies was used to assess the quality of included studies and to assess the risks of biases. The quality of included studies was independently appraised by two reviewers (AT and AH). (1) appropriate sampling frame, (2) proper sampling technique, (3) adequate sample size, (4) study subject and setting description, (5) sufficient data analysis, (6) use of valid methods for the identified conditions, (7) valid measurement for all participants, (8) using appropriate statistical analysis, and (9) adequate response rate are the 9 parameters of the assessment tool [17].

If none of the parameters were met, a score of 1 was assigned. We agreed to rate an item as 1 when the information provided was insufficient to assist us in making a decision (failure to satisfy a specific item). Bias risks were categorized as low (total score, 0 to 2), moderate (total score, 3 or 4), or high (total score, 5 to 9). Finally, this review included articles of low and moderate risk of biases (S3 File).

### Statistical methods and analysis

The null hypothesis of no substantial heterogeneity across studies was tested using Cochran’s Q test. The weighted sum of squared differences between individual study effects and the pooled effect across studies, with the weights being those used in the pooling procedure, is used to calculate Cochran’s Q [18]. The chi-squared distribution of Cochran’s Q statistic has k—1 degree of freedom, where k is the number of studies. In the statistical heterogeneity test of Cochran’s Q, P < 0.05 is considered statistically significant.

Because the percentage of variation in the measures of association across trials is due to heterogeneity rather than chance, the I^2^ statistic was also calculated. I^2^, which is equal to the quantity of Cochran’s Q minus its degree of freedom (df) divided by Cochran’s Q times 100% (100% *(Q − df)/Q), has values ranging from 0% to 100%, with 0% indicating no observed heterogeneity and large numbers indicating increasing heterogeneity. Low, moderate, and high heterogeneity are defined as I^2^ values of 25, 50, and 75 percent, respectively [19]. The test statistic revealed high heterogeneity among the included studies in this review (I^2^ >= 97.23%, p 0.001). As a result, Der Simonian and Laird’s pooled effect was estimated using a random-effects model.

Egger’s weighted regression and Begg’s rank correlation tests were used to look for publication bias (P<0.05 is considered statistically significant). Random-effects meta-analysis was conducted to combine the findings of the included studies, and the results were reported as proportions of safe food handling practice and associated factors with 95% confidence intervals. The influence of a single study on the total pooled estimate was assessed using a sensitivity analysis utilizing a random-effects model. Meta-regression was used to determine the likely source of heterogeneity, with the sample size and year of publication as input data. Neither of them, however, was statistically significant (p>0.05). The combined effect was expressed as an odds ratio.

## Results

### Description of included studies

**Fig 1:**
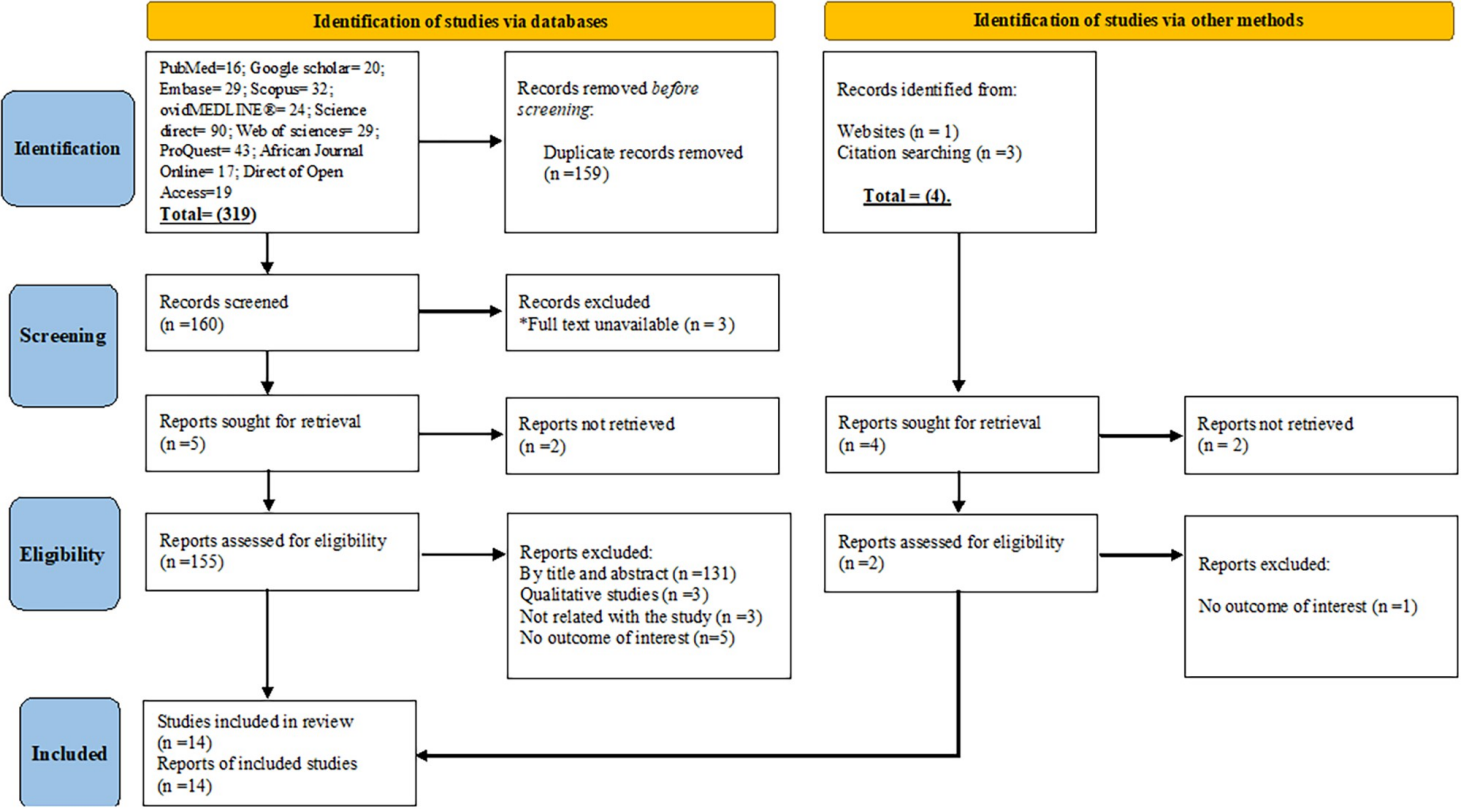
PRISMA flowchart for the selection of eligible studies on food handling practice among food handlers in foodservice establishments of Ethiopia, 2022.

In total, 323 articles were identified from (PubMed (n = 16), Google Scholar (n = 20), African Journal Online (n = 17), Embase (n = 29), Science Direct (n = 90), ProQuest (n = 43), Direct of Open Access Journals (n = 19), ovidMEDLINE^®^ (n = 24), Web of Sciences (n = 29), and other sources (n = 4)), with 159 duplicates removed. We screened the titles and abstracts of 164 articles, from which 14 met the criteria for inclusion and were included in the final analysis (Fig 1).

### Characteristics of included studies

As shown in Table 1, 14 studies met the criteria for inclusion. By design, all of the studies included were cross-sectional. The current systematic review and meta-analysis included a total of 4849 food handlers. The included studies had a median response rate of 98.5%. The studies were published between 2014 through 2022. The sample size in this meta-analysis ranged from 116 to 430. The respondents’ mean age ranged from 24.9±1.3 to 37.7±1.5 years. In this review, a study conducted in the Somali region had the lowest proportion of safe food handling practices (20.9%), while a study conducted in the Amhara region had the highest (72.4%).

**Table 1:** Baseline characteristics of the included studies, 2014–2022.

Authors’ Name& Publication Year	Study Area	Study Region	Study Setting	Design	Sampling	Sample Size	Response rate	Safe practice	Risk of bias
Chekol et al., 2019 [15]	Debark Town	Amhara	Cafes, restaurants, butcheries	CS	Srs	422	98.6%	40.1%	Low
Reta et al., 2021 [20]	Woldia Town	Amhara	Cafés and restaurants	CS	SRS	288	100%	46.5%	Moderate
Tessema et al., 2014 [21]	Dangila Town	Amhara	Hotel, cafes, juice shops, restaurants	CS	SRS	430	94.4%	52.5%	Low
Alemayehu et al., 2021 [13]	Debre Markos Town	Amhara	Cafes, hotels, restaurants	CS	Srs	408	100%	53.7%	Low
Teferi et al., 2021 [22]	Fiche Town	Oromia	Restaurants, juice houses, butcher shops	CS	SRS	422	98.9%	50.5%	Low
Derso et al., 2017 [23]	Bahirdar Town	Amhara	Restaurants	CS	Srs	417	98.8%	67.6%	Moderate
Legese et al., 2017 [24]	Arba Minch Town	SNNPR	Cafes, hotels, restaurants	CS	Srs	387	98.7%	32.5%	Moderate
Mohamed, 2021 [25]	Godey Town,	Somalia	Cafes, hotels, restaurants	CS	SRS	390	98.2%	20.9%	Low
Abdi et al., 2020 [11]	Bole Sub-city	Addis Ababa	Hotels, cafés bars, Coffee Shops	CS	SRS	394	95.17%	27.4%	Low
Tesfaye et al., 2020 [26]	Shashemane Town	Oromia	Cafés, canteens	CS	srs	120	100%	27.5%	Low
Azanaw et al., 2019 [14]	Gondar City	Amhara	Hotels and restaurants	CS	srs	384	100%	49.0%	Low
Adane et al., 2018 [12]	Dessie Town	Amhara	Street food shops, hotels	CS	srs	116	100%	72.4%	Low
Lalit et al., 2015 [27]	Mekele Town	Tigray	Restaurants, cafes, juice houses	CS	SRS	369	97%	53.1%	Low
Abe et al., 2021 [28]	Batu Town	Oromia	Hotels	CS	SRS	302	100%	58.0%	Low

CS: Cross-sectional.

SRS: Systematic random sampling.

srs: simple random sampling.

SNNPR: Southern Nations Nationalities and Peoples’ Region.

The 14 studies were conducted across five regions and 1 self-administered city. Three investigations were conducted in the Oromia region, seven in Amhara, one in Tigray, one in the South Nations and Nationalities and Peoples’ Region (SNNPR), one in Somalia, and one in Addis Ababa city. While the majority of the studies used interviews and questionnaires to gather data, five investigated hygiene and sanitation conditions through inspection also. When it comes to the quality of the studies, the majority (64.3%) had a low risk of bias. Additionally, all investigations were subjected to further analysis to uncover contributing factors to safe practice (Table 1).

### Meta-analysis of pooled safe food handling practices

**Fig 2:**
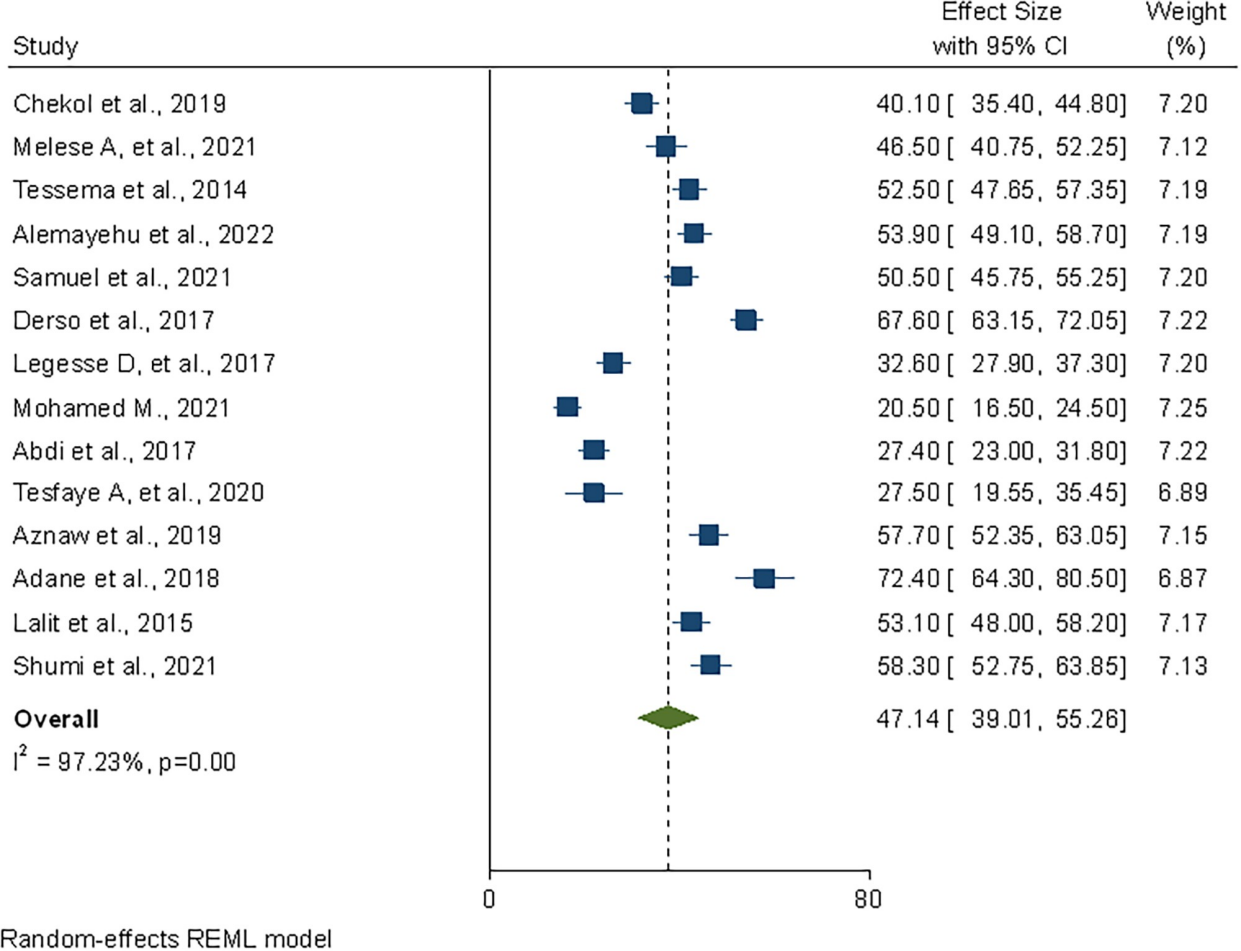
Forest plot depicting pooled prevalence estimate of safe food handling practices among food handlers in foodservice establishments of Ethiopia, 2022.

The prevalence estimate varied among studies with significant heterogeneity (P < 0.001; I^2^ = 97.23%) As a result, we employed a random effect model. The pooled prevalence estimate of safe food handling practice among Ethiopian food handlers was 47.14% (95% CI: 39.01–55.26) based on the DerSimonian–Laird random-effect model. A forest plot depicts the prevalence estimates of food handling practice among food handlers (Fig 2).

### Subgroup analysis

**Fig 3:**
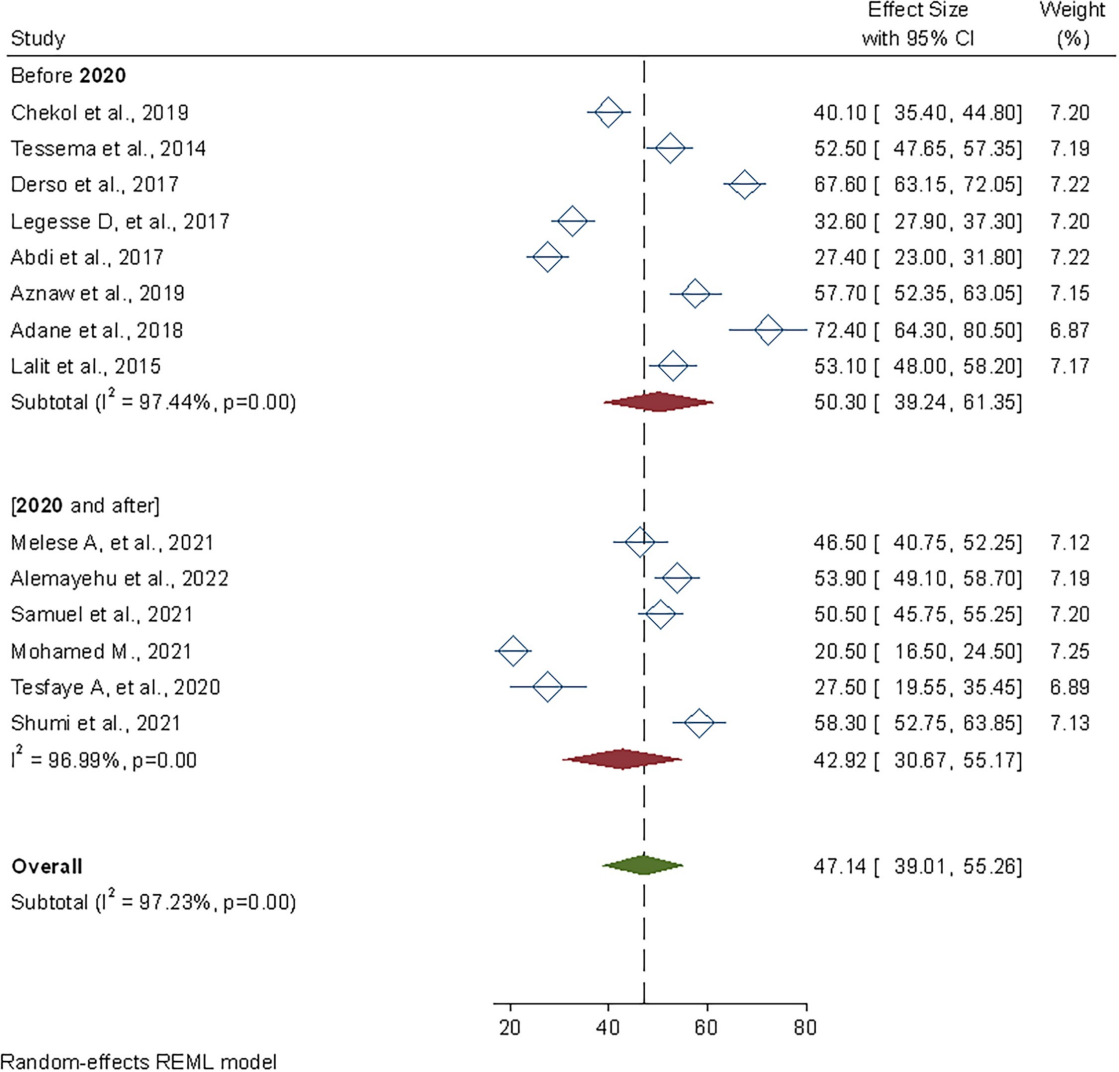
Sub-group pooled prevalence estimate of safe food handling practices by study year among food handlers in foodservice establishments of Ethiopia, 2022.

We performed a subgroup analysis based on the year of publication of the studies in this meta-analysis. Accordingly, the pooled prevalence of safe food handling practice was 50.3% (95% CI: 39.26–61.35) for studies conducted before 2020, but it exhibited a drop for studies conducted after the Coronavirus disease (COVID-19), with a pooled prevalence of 42.92% (95% CI: 30.67–55.17) (Fig 3).

**Fig 4:**
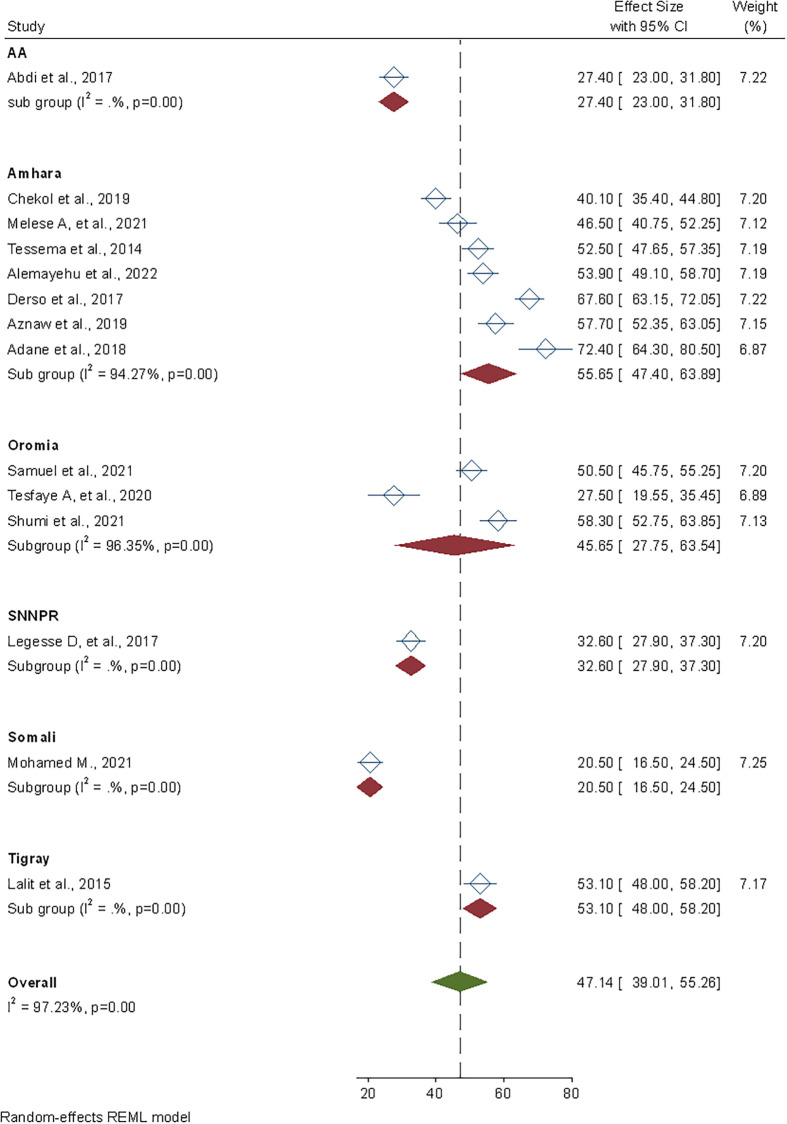
Sub-group pooled prevalence estimate of safe food handling practices by region among food handlers in foodservice establishments of Ethiopia, 2022.

Similarly, a subgroup analysis based on geographical location (country region) was undertaken to see if there were any regional differences in safe food handling methods. As a result, the Amhara 55.6% (95% CI: 47.40–63.89) and Somali 20.50% (95% CI: 16.50–24.50) regions had the highest and lowest prevalence of safe food handling practice, respectively (Fig 4).

### Heterogeneity and publication bias

**Fig 5:**
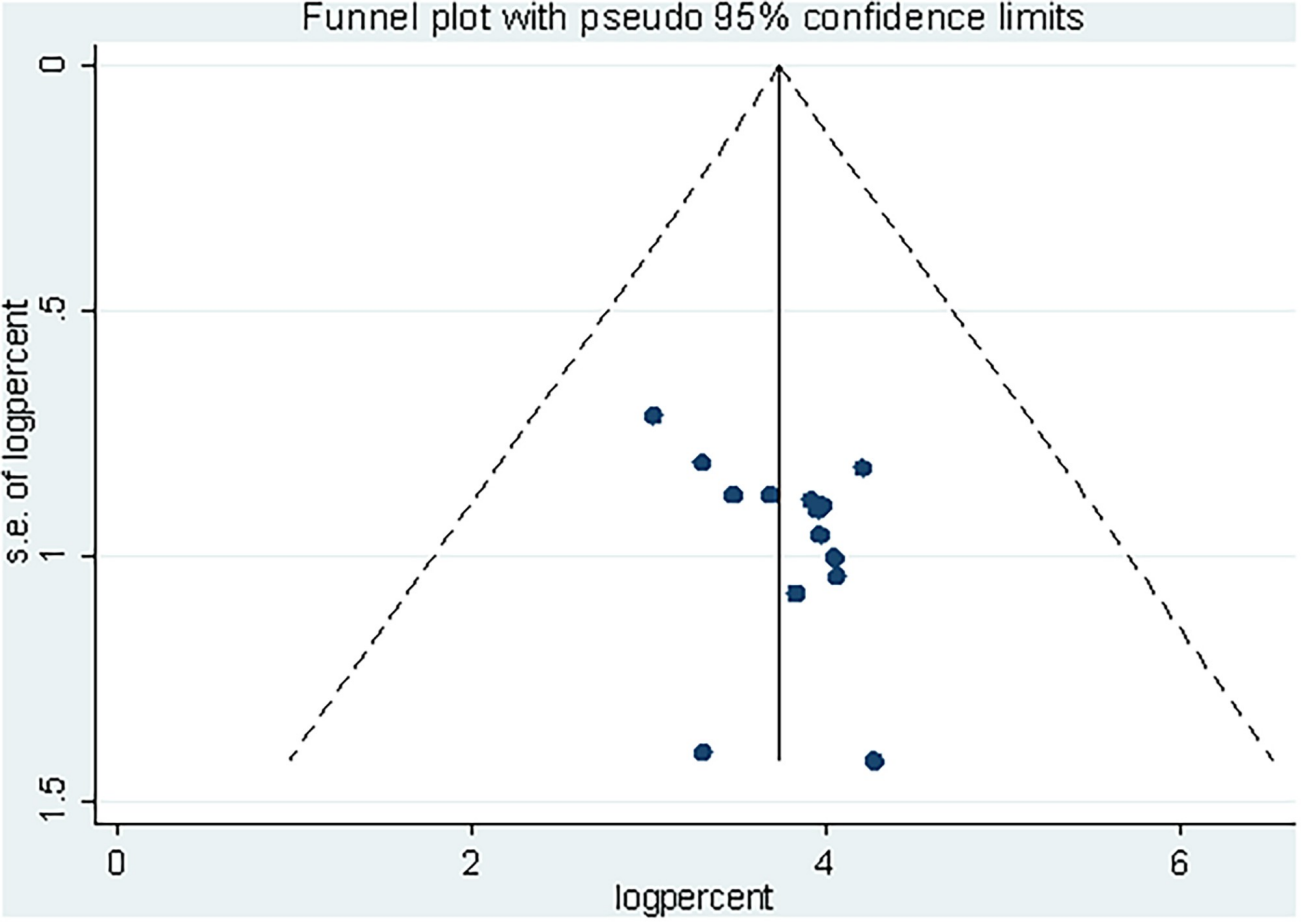
Funnel plot showing publication bias of food handling practice studies among food handlers in public food establishments of Ethiopia, 2022.

Visual inspection of the symmetrical funnel plot (Fig 5) revealed no publication bias, which was statistically supported by Begg’s test (P = 0.314) and Egger’s test (bias coefficient (B) = 6.13 (95%CI = − 4.07–12.98; P = 0.378). As a result, we did not use the Duval and Tweedie non-parametric/ trim and fill method to fill in missing theoretical studies.

In addition, a univariate meta-regression with sample size and publication year was performed for possible heterogeneity. The results of the analysis show that neither of the two had a significant impact on study heterogeneity (Table 2).

**Table 2:** Univariate meta-regression of factors related to the heterogeneity of food handling practices among food handlers at food service establishments in Ethiopia, 2022.

Variables	Coefficient	Standard Error	P-value
Sample size	-0.01879	.0434127	0.6637
Year	-.911937	1.818519	0.616

### Sensitivity analysis

Sensitivity analysis using the random-effects model revealed that no single study influenced the overall prevalence of safe food handling practices among food handlers (Table 3).

**Table 3:** Sensitivity analysis for estimates on safe food handling practice among food handlers in Ethiopia, 2022.

Authors’ Name*	Prevalence (%)	95%CI	I^2^ (%)	Heterogeneity chi-squared (Q)	p-value
Chekol et al., 2019	47.69	38.81–56.56	97.47	473.56	<0.001
Reta et al., 2021	47.19	38.39–55.98	97.49	478.81	<0.001
Tessema et al., 2014	46.72	37.90–55.55	97.45	470.11	<0.001
Alemayehu et al., 2021	47.14	38.92–55.35	97.29	478.95	<0.001
Teferi et al., 2021	46.88	38.00–55.76	97.47	474.20	<0.001
Derso et al., 2017	45.53	37.60–53.47	96.79	374.05	<0.001
Legesse et al., 2017	48.27	39.63–56.90	97.32	447.75	<0.001
Mohamed, 2021	49.21	41.90–56.52	96.14	370.65	<0.001
Abdi et al., 2020	48.67	40.38–56.96	97.06	407.76	<0.001
Tesfaye et al., 2020	48.59	40.11–57.07	97.38	458.82	<0.001
Azanaw et al., 2019	48.58	40.38–56.79	97.21	458.82	<0.001
Adane et al., 2018	45.27	37.02–53.52	97.24	435.16	<0.001
Lalit et al., 2015	46.88	37.90–55.46	97.49	469.62	<0.001
Abe et al., 2021	46.28	37.67–54.89	97.37	457.01	<0.001

*Given study name is omitted.

### Factors associated with safe food handling practice

**Fig 6:**
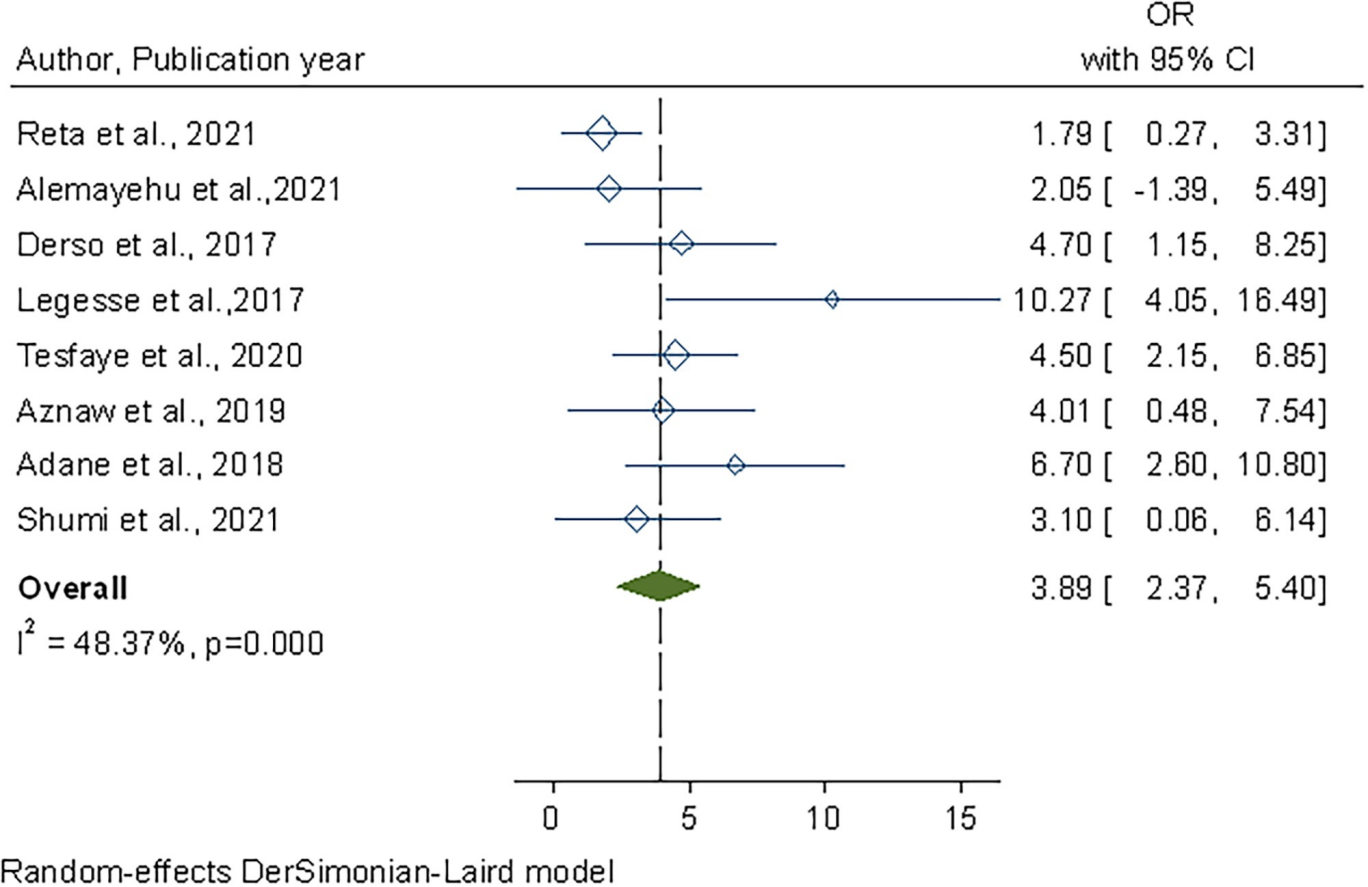
Forest plot of the association between training and safe food handling practices in Ethiopia, 2022.

Eleven studies with eight factors were included in the meta-analysis of factors associated with safe food handling practices, (S5 File). Food handlers who had received service training were 3.89 times more likely than those who had not received training to have safe food handling practice (OR, 3.89; 95% CI: 2.37–5.40) (Fig 6).

**Fig 7:**
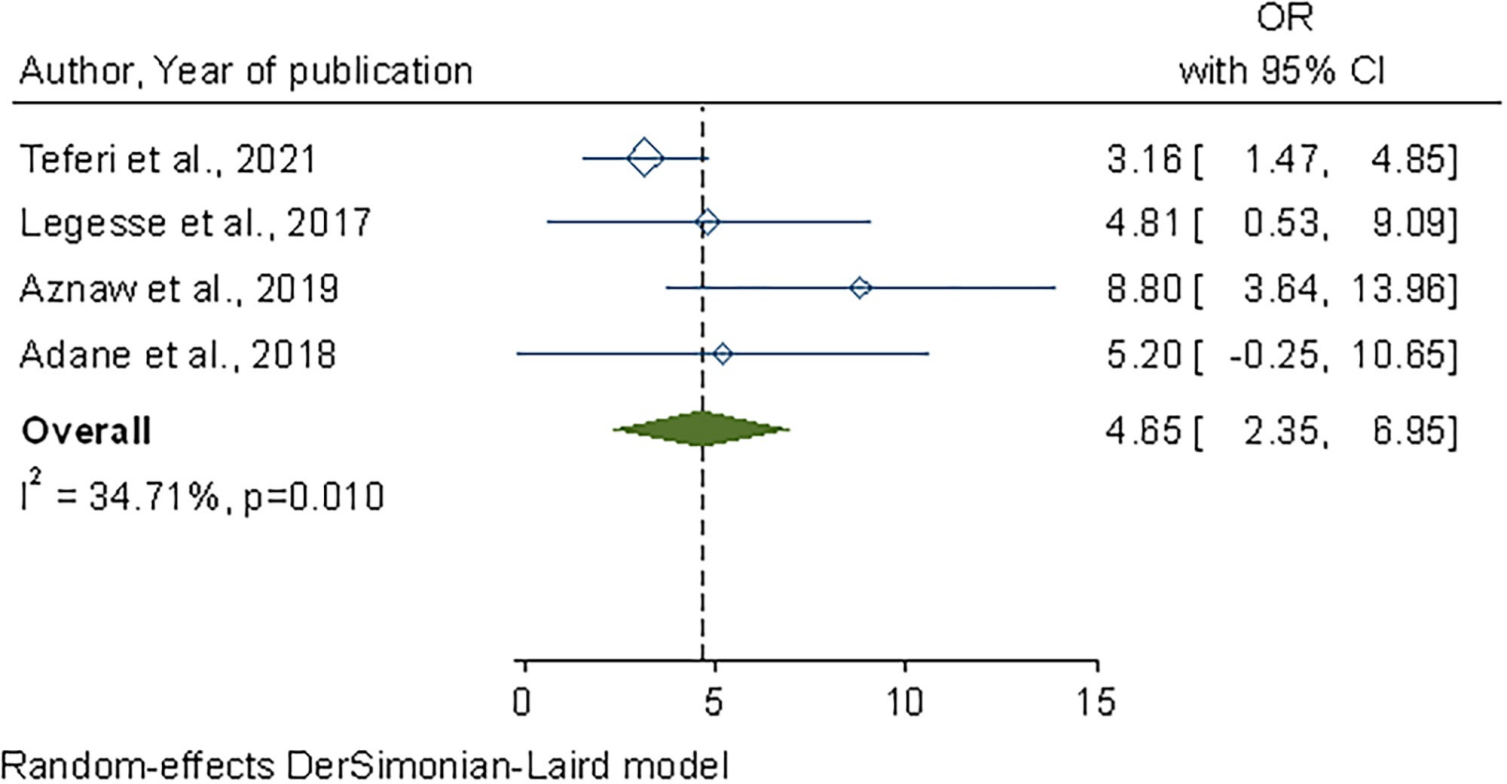
Forest plot of the association between availability of an on-site water storage facility and safe food handling practices in Ethiopia, 2022.

Similarly, food handlers who worked in establishments with on-site water storage facilities were 4 times more likely to have safer practices than those who worked in establishments without water storage tanks. (OR, 4.65; 95% CI: 2.35–6.95) (Fig 7).

**Fig 8:**
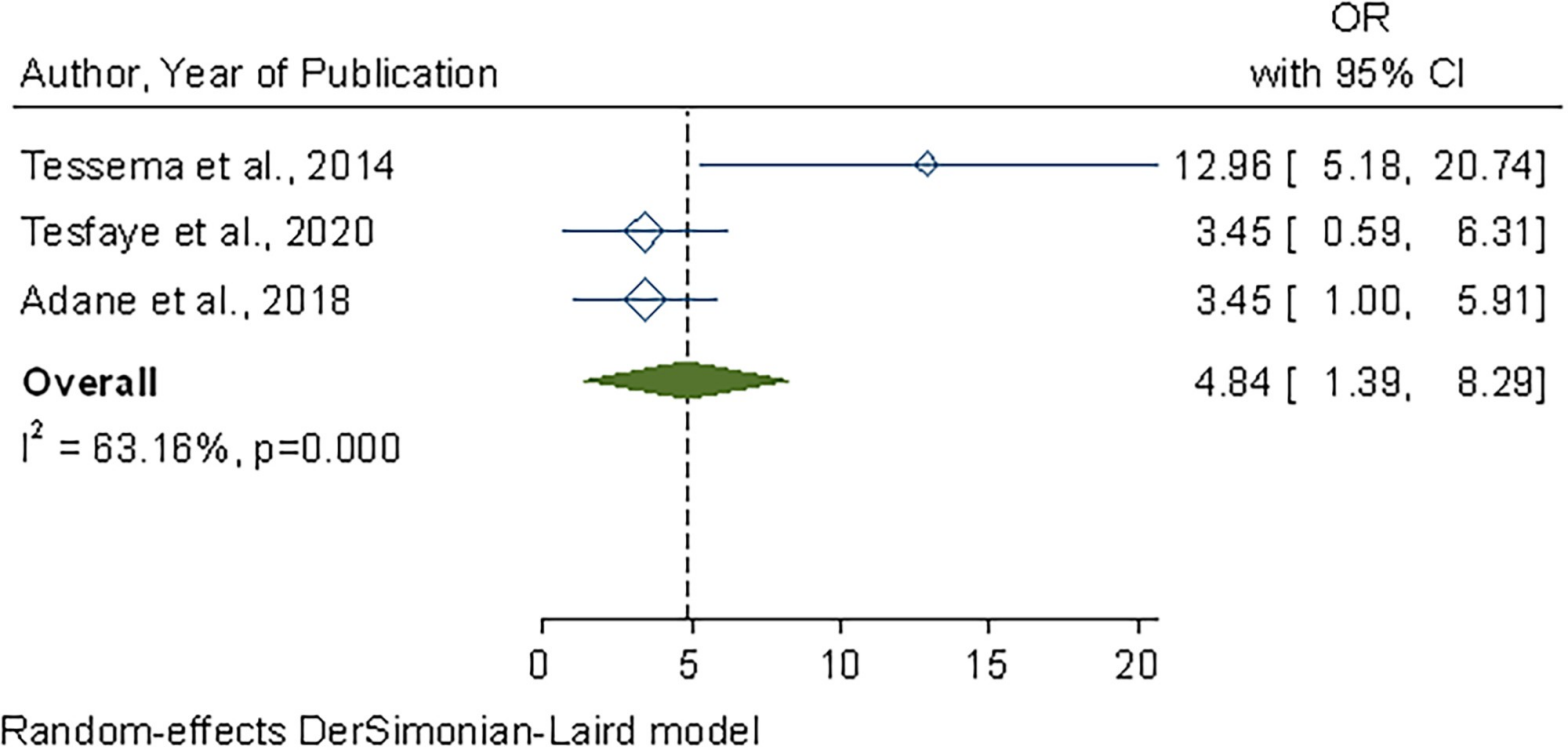
Forest plot of the association between attitude and safe food handling practices in Ethiopia, 2022.

A favourable food handling attitude was associated with safe food handling practice. Food handlers with a positive attitude toward food safety were 4.8 times more likely to have a safer practice. (OR, 4.89; 95% CI: 1.39–8.29) (Fig 8).

**Fig 9:**
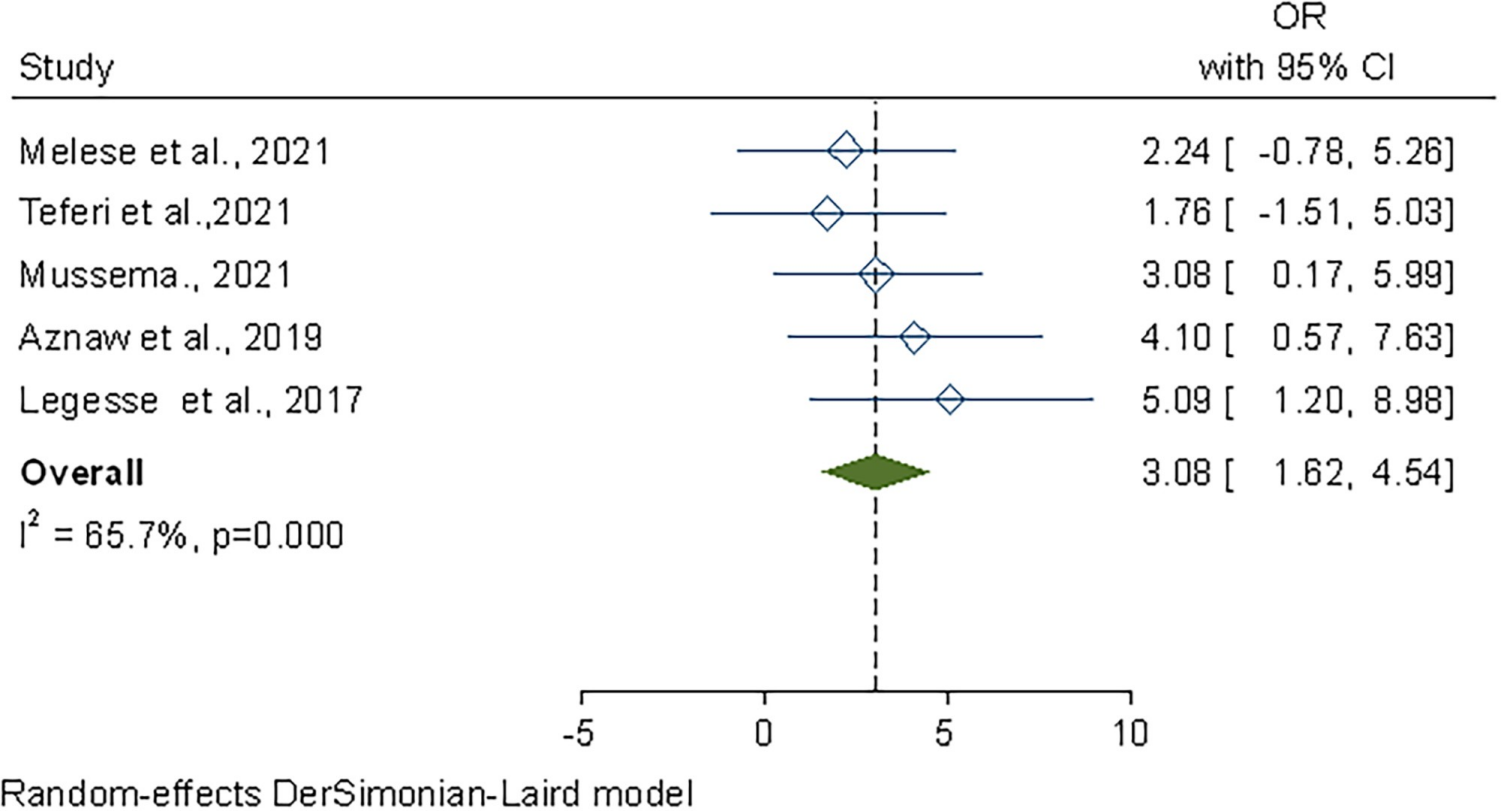
Forest plot of the association between sanitary inspection and safe food handling practice in Ethiopia, 2022.

Finally, food handlers who worked in places that were accredited with hygiene and sanitary inspections were 3 times more likely to have a safer practices than their counterparts who worked in establishments that didn’t have certification (OR, 3.08; 95% CI: 1.62–4.45) (Fig 9).

## Discussion

Food businesses have the potential to severely affect human health if they do not adhere to food safety recommendations. Nearly seventy-five percent of food-borne illness outbreaks at public food establishments are thought to be linked to employees’ improper food handling practices [29]. Serious foodborne disease outbreaks have occurred on every continent in the past decade, typically exacerbated by poor food handling [30]. As a result, the current findings provide relevant insight into Ethiopia’s recent food safety practice, its geographical distribution, and associated factors.

In this meta-analysis, the estimated prevalence of safe food handling practices among Ethiopian food handlers was 47.14% (95% CI: 39.01–55.26). The pooled proportion was lower than the 54.7% recorded in Malaysia and Nigeria [31, 32], notwithstanding the dearth of systematic reviews or meta-analyses on related themes in Ethiopia or elsewhere. This shows that there are significant differences in food safety practice across countries, which could be due to social, cultural, and/or environmental differences. Ethiopia is also one of the world’s least developed countries, with a less organized food safety system. Population growth, urbanization, environmental concerns, and food hygiene concerns continue to place strain on the country’s food systems, compromising food quality and safety [33].

The pooled prevalence of safe food handling practice in studies conducted before 2020 was 50.3%, while it was 42.92% in studies conducted after 2020. As a result of pandemic-driven changes in safety requirements, food business operators’ jobs have become significantly more challenging. In addition to continuing to follow existing food safety and FBD control measures (such as keeping the premises and one’s self clean, separating raw and cooked meals, thoroughly cooking, keeping food at safe temperatures, and using safe water and raw materials, etc.), [34] hand sanitizers at entryways, personnel wearing masks and gloves, and tables placed six feet apart are the top three customer and regulatory expectations from food establishments in the COVID era [35].

Most businesses in resource-poor environments fail to accomplish this because they rely on the same supply chains as businesses in wealthier countries, but have far less negotiating power [36]. Moreover, as the pandemic unfolded, the implementation of inspection operations, in particular, is believed to have slowed. Although Ethiopian food handlers were required to get tested for COVID-19, there were no extra inspections to assess adherence to additional food safety standards (carrying a food handler’s certificate, wearing protective clothing, having access to running water, disposing of waste, etc…) [37, 38].

In many circumstances, the consequences of food handling can be traced back to a range of dynamic, complicated interactions between humans and their surroundings [39]. There were considerable regional differences in food handling practice across Ethiopia in this meta-analysis. The highest and lowest prevalence of safe food handling practice were found in the Amhara (55.6%) and Somali (20.50%) regions, respectively. Because of the high prevalence of poverty and social indicators that lag far behind national averages, the Ethiopian government has designated the Somali region as a developing regional state [40]. While differences in prevalence between regions may be due to differences in socio-demographic characteristics and the number of studies included in each category of analysis, what is known is that some of the major challenges in terms of food safety revolve around inadequacies, inconsistencies, inequities, and inefficiencies [39].

When it comes to food safety in food businesses, the responsibility is solely on the establishment. Food service operators must ensure that food handlers receive adequate supervision and food hygiene training that is appropriate for the area they work in [41]. Food handlers who had received service training in Ethiopia were 3.8 times more likely to follow safe food handling recommendations in this review. This is in line with the findings of a Systematic Review and Meta-Analysis of the impact of food safety and hygiene training on food handlers, which revealed that training enhances food handlers’ safe practice [42]. Additionally, while training is crucial for public health, it is also critical for a food business’s long-term commercial success. Training helps to support businesses in being more efficient, competitive, and lucrative; it boosts performance standards, promotes the company image, and increases employee morale while decreasing waste [43]. Therefore food handlers must get effective and frequent food safety training as a first step in guaranteeing that, food safety concepts are at least introduced.

When we think of food safety, there is no business in the food service industry that does not rely on potable water for its basic operations [44]. Water is a significant epidemiological determinant of foodborne infections. Lack of access to improved water sources, insufficient peak demand quantity, and pressure maintenance issues can all have a significant impact on food hygiene and preparation [45]. One-third of Ethiopia’s population lacks access to safe drinking water. Another 28% have limited access, which means the water is likely safe but collecting it takes more than 30 minutes owing to distance from the source, long lines, or both. This creates a need for storage tanks [46, 47].

In this meta-analysis, food handlers who worked in establishments with on-site water storage tanks were four times more likely to have safer practices than those who didn’t. This finding was supported by two studies conducted in Ethiopia and Jordan[24, 48], which found that ongoing access through storage tanks, improved safe handling. However, more research is needed to identify the user practices that improve water quality, the tank/vessel material that is best suited for bacteriological and physiochemical contamination, and the water retention period in tanks.

If the foodservice community does not see the situation as a big public health threat, food-borne diseases may be difficult to control and eventually eliminate [49]. Food handlers should have the necessary information, set realistic expectations, and put in place the necessary preventative and control measures to do this [50]. The context of safe food handling is determined by one’s attitude toward food safety. Attitude formation, or the learned tendency to think, feel, and act in a certain way toward a set of items, has been proven to be an important motivator for behavior adoption [51].

People who are more concerned about FBD origins, the prevalence and frequency of severe episodes, mitigation strategies, and economic and health-related repercussions engage in more protective activities [49]. In keeping with this, the current meta-analysis found that having a positive attitude towards food handling was associated with safe food handling practices. Education and training can assist in overcoming attitudinal barriers to safe practice [42]. Thus, if food handlers are consistently encouraged to follow recommendations and are informed about their universal susceptibility to FBDs, their attitudes toward food safety may improve.

Food safety inspections have traditionally included evaluations of food handling methods and the state of food preparation facilities. A safety inspection is a tool for establishing transparency and driving market pressure through the creation of compliance incentives [52]. Because inspections are normally conducted within a regulatory framework, compliance motivators such as public disclosure of inspection scores or reports, closures, monetary fines, and other enforcement measures can help to ensure that standard operating procedures are being followed [53]. This was also the case in the present meta-analysis, food handlers who worked in facilities with hygiene and sanitary inspection certificates were three times more likely to adopt safer practices.

Within Ethiopia, however, the approach to food safety inspection differs greatly between jurisdictions. Some jurisdictions create a framework of prescriptive food safety requirements for food businesses, using inspection to assess the extent to which certain criteria are being met. Other jurisdictions, on the other hand, examine food handling practices and environments using a qualitative risk assessment, which is often guided by established risk assessment frameworks such as Hazard Analysis and Critical Control Point Planning (HACCP) or other proprietary risk assessment frameworks [54]. Future research is needed to harmonize the meanings given to food safety inspection across consumers, food business operators, and food safety inspectors in different parts of Ethiopia, as well as concepts including compliance, in the application of food safety inspection.

This review has some limitations. First, only articles published in English were included in the search. Second, the investigations were all observational, and qualitative approaches did not back the findings. Finally, only five Ethiopian regions and one administrative city were covered in this meta-analysis

### Future scope

Due to limited resources, gathering data on the number of consumers who fall ill as a result of diseases possibly caused by unsafe practices is challenging. However, whenever practicable, efforts should be made to collect this data. To inform managers and policymakers, a review of the expenses associated with unsafe practice-related diseases and worker and process downtime as a result of FBDs should be conducted. Similarly, there seems to be a need for future research to identify barriers to the implementation of safe food handling practices. Other potential research areas include issues related to food handlers who work in food establishments working outside of legitimate businesses. This may provide a more complete picture of the industry

Future studies should also take into account the microbiological quality of foods served in such environments. Latly, while the current study identified training as an important determinant of safe practice, it did so in the context of the larger picture. Future research may need to deconstruct it, even more, looking at the type and frequency of training, as well as the content delivered.

## Conclusion

Food-borne diseases continue to be a global concern, resulting in high morbidity and mortality as well as significant financial expenditures. This review identified that improvements are needed in food handling training, government regulation, and infrastructure. Standard regulations for food service establishments must be implemented, on a local and national level. Licensure requirements that are strictly enforced and the public provision of more comprehensive free courses that address food-handling practices in such venues are examples of such regulation. Though regulation and training are critical for long-term food safety, failing to address infrastructure issues can jeopardize public health efforts. Access to safe water and the existence of sanitary waste management should all be part of the basic infrastructure for preserving food business safety.

## Supporting information

S1 File PRISMA checklist. (DOC)

S2 File Literature search strategies. (DOCX)

S3 File Quality appraisal checklist. (DOCX)

S4 File Operational definition of included studies. (DOCX)

S5 File Extracted data. (XLSX)

## Abbreviations

CI: Confidence Interval
FBDs: Foodborne diseases
HACCP: Hazard Analysis and Critical Control Point Planning
JBI: The Joanna Briggs Institute
LMICs: low- and middle-income countries
OR: Odds Ratio
PRISMA: Preferred Reporting Items for Systematic Reviews and Meta-Analyses
SNNPR: Southern Nations Nationalities and Peoples’ Region
