# Supplementary material for: Food safety practice and associated factors in public food establishments of Ethiopia: A systematic review and meta-analysis
Source: PLoS One. 2022 May 27;17(5):e0268918. doi: 10.1371/journal.pone.0268918 (PMC9140249; doi:10.1371/journal.pone.0268918)
Supplement: S2 File — (DOCX) [file pone.0268918.s002.docx]

Supplementary file 2: Table S1. Literature Search strategies for food safety among food handlers of food service establishments in Ethiopia, February 05, 2022

| S.N. | Database | Search strategy | Search results |
| --- | --- | --- | --- |
| 1 | PubMed | ((("Food Safety"[MeSH Terms] OR "Food hygiene"[All Fields] OR "Food sanitation"[All Fields] OR "Food handling"[All Fields]) AND "Practice"[All Fields] AND "Associated factors"[All Fields]) OR "related factors"[All Fields] OR "Determining factors"[All Fields]) AND ("Food handler"[All Fields] OR "Food handlers"[All Fields]) AND ("Ethiopia"[MeSH Terms] OR "Ethiopia"[All Fields] OR "Ethiopia’s"[All Fields]) | 16 |
| 2 | Google scholar | allintitle: Ethiopia "Food safety practice" OR "Food hygiene practice" OR "Food sanitation practice" OR "Food handling practice" OR "Food safety practice" OR "Food sanitation practice" OR "Food handlers practice" | 20 |
| 3 | Embase | ('food handlers' OR (('food'/exp OR food) AND handlers)) AND ('ethiopia'/exp OR ethiopia) | 29 |
| 4 | Scopus | TITLE-ABS-KEY ( ''food AND handlers'' AND ethiopia ) | 32 |
| 5 | ovidMEDLINE® | (Food handlers and Ethiopia).mp. [mp=title, abstract, original title, name of substance word, subject heading word, floating sub-heading word, keyword heading word, organism supplementary concept word, protocol supplementary concept word, rare disease supplementary concept word, unique identifier, synonyms] | 24 |
| 6 | Science direct | Find articles with these terms: Food handlers and Ethiopia(Research articles highlighted)= | 90 |
| 7 | Web of sciences | TOPIC: (Food handlers and Ethiopia) | 29 |
| 8 | ProQuest | ab(food handlers) AND ab(Ethiopia) | 43 |
| 9 | African Journal Online | “food handlers" AND "Ethiopia” | 17 |
| 10 | Direct of Open Access Journals | “Food handlers" AND "Ethiopia” | 19 |
|  | Total |  | 319 |
