# Supplementary material for: Food safety practice and associated factors in public food establishments of Ethiopia: A systematic review and meta-analysis
Source: PLoS One. 2022 May 27;17(5):e0268918. doi: 10.1371/journal.pone.0268918 (PMC9140249; doi:10.1371/journal.pone.0268918)
Supplement: S3 File — (DOCX) [file pone.0268918.s003.docx]

**Supplementary file 2**

**Table S2: Individual quality assessment of 14 articles included in the review of the status of food handling practice among food handlers in Ethiopia, studies published between 2014 and 2022.**

| **Authors, Year** | **Sampling frame** | **Sampling strategy** | **Sample size** | **Description of research setting & population** | **Data analysis conducted with sufficient coverage** | **Valid methods used for the identification of the condition** | **Reliability of the instrument used** | **Statistical analysis methods** | **Response rate** | **Total** | **Risk of bias** |
| --- | --- | --- | --- | --- | --- | --- | --- | --- | --- | --- | --- |
| Chekol et al., 2019 | 0 | 0 | 0 | 0 | 0 | 1 | 1 | 0 | 0 | 2 | Low |
| Reta et al.,  2021 | 1 | 0 | 0 | 0 | 0 | 1 | 1 | 1 | 0 | 4 | Moderate |
| Tessema et al., 2014 | 0 | 0 | 0 | 0 | 0 | 1 | 1 | 0 | 0 | 2 | Low |
| Alemayehu et al., 2022 | 0 | 0 | 0 | 0 | 0 | 1 | 1 | 0 | 0 | 2 | Low |
| Teferi et al.,2021 | 0 | 0 | 0 | 0 | 0 | 1 | 1 | 0 | 0 | 2 | Low |
| Derso et al., 2017 | 0 | 0 | 0 | 0 | 0 | 1 | 1 | 1 | 0 | 3 | Moderate |
| Legese et al.,2017 | 1 | 1 | 0 | 0 | 0 | 1 | 1 | 0 | 0 | 4 | Moderate |
| Mohamed,  2021 | 0 | 0 | 0 | 0 | 0 | 0 | 0 | 0 | 0 | 0 | Low |
| Abdi et al.,  2017 | 0 | 0 | 0 | 0 | 0 | 1 | 1 | 0 | 0 | 2 | Low |
| Tesfa et al.,2020 | 0 | 0 | 0 | 0 | 0 | 1 | 1 | 0 | 0 | 2 | Low |
| Aznaw et al., 2019 | 0 | 0 | 0 | 0 | 0 | 1 | 1 | 0 | 0 | 2 | Low |
| Adane et al., 2018 | 0 | 0 | 0 | 0 | 0 | 1 | 1 | 0 | 0 | 2 | Low |
| Lalit et al., 2015 | 0 | 0 | 0 | 0 | 0 | 1 | 1 | 0 | 0 | 2 | Low |
| Shumi et al.,2021 | 0 | 0 | 0 | 0 | 0 | 1 | 1 | 0 | 0 | 2 | Low |
