# Supplementary material for: Food safety practice and associated factors in public food establishments of Ethiopia: A systematic review and meta-analysis
Source: PLoS One. 2022 May 27;17(5):e0268918. doi: 10.1371/journal.pone.0268918 (PMC9140249; doi:10.1371/journal.pone.0268918)
Supplement: S4 File — (DOCX) [file pone.0268918.s004.docx]

Table S3: Operational definitions used for the outcome of 14 articles included in the review of the status of food handling practice among food handlers in Ethiopia, studies published between 2014 and 2022.

| Author | Operational Definition (Safe food handling practice) | Data Collection method |
| --- | --- | --- |
| Chekol et al., 2019 | Food handling practice was computed by using 21 questions and 29 observational check-lists totally 50 questions. Accordingly, participants who respond 70% and above considered as having good food handling practice whereas those who respond below 70% considered as having poor food handling practice. | Interview administered questionnaire & Observation |
| Melese et al., 2021 | The good handling practices of food handlers were assessed and evaluated based on observational checklists. The section had eighteen questions/statements. Each correct practice reported scored one point. For evaluation, a score >50% by an individual was considered as having "good/ or adequate" FHPs. | Observation |
| Tessema et al., 2014 | To assess the level of Practices, respondents were asked 17 questions from the questionnaire and those who scored ≤ the mean value were considered as having poor Practices and those who scored > the mean value were considered as having good Practices | Interview administered questionnaire and  observational checklist |
| Alemayehu et al., 2022 | Food handling practice was assessed by using an observational checklist during serving (working). These practices were also scored and one mark was given for every standard practice and zero for every unhygienic practice. Food-handlers that obtained total score ≥ mean were considered to have “safe practice” and those that had scores < mean were considered to have “unsafe practice | Observation |
| Samuel et al., 2021 | To assess the level of Practices, respondents were asked 19 questions from the questionnaire and those who scored ≤ the mean value were considered as having poor Practices and those who scored > the mean value were considered as having good Practices | Interview administered questionnaire |
| Derso et al., 2017 | The level of food hygiene practice was determined by using 17 food hygiene practice questions complemented with direct observation. The food hygiene practice was computed with a maximum score of seventeen. By considering the mean score (12), the food hygiene practice of food handlers was categorized as poor if their score was below twelve, otherwise good practice if their score was greater or equal to twelve | Interview administered questionnaire + observational checklist |
| Legesse et al., 2017 | Food handling practices: To assess the level of Practices, respondents were asked 17 questions from the questionnaire and those who scored ≤ the mean value were considered as having poor Practices and those who scored > the mean value were considered as having good Practices | Interview administered questionnaire |
| Mohamed M., 2021 | Food safety practice was assessed based on 12 food safety practice related questions which contain three options always, sometimes and never. Correct answer was given 2, sometime was given one and incorrect answer was given zero. Total score was range from (0-24). Food handlers score 17 and above were considered as good, whereas food handlers score 16 and below 16 were considered as poor practice | Interview administered questionnaire |
| Abdi et al., 2017 | Food hygiene practice level: respondents who scored less than 80% of their response to the total (16) food hygiene practice-related questions were considered as having poor level of practices. Those who scored equal to or more than 80% were considered as having a good level of practice. | Interview administered questionnaire |
| Tesfaye A, et al., 2020 | The level of food hygiene practice was determined by using 17 food hygiene practice questions complemented with direct observation. The food hygiene practice was computed with a maximum score of seventeen. By considering the mean score (12), the food hygiene practice of food handlers was categorized as poor if their score was below twelve, otherwise good practice if their score was greater or equal to twelve | Interview administered questionnaire + observational checklist |
| Aznaw et al., 2019 | Food handling practice: food handlers were asked seventeen questions and those who scored less than or equal to the mean value were considered as having poor practice and those who scored greater than the mean value were considered as having good practice | Interview administered questionnaire |
| Adane et al., 2018 | Food handlers' level on food hygiene and safety measures was set using 14 questions. For the descriptive statistics of level of food hygiene and safety, three categories were considered, i.e., poor: _60%, good: 60%±80%, and very good: 80%±100%. For further analyses, food handlers who scored _9 points were considered to have a good level of food hygiene and safety practice, whereas food handlers who scored *<*9 points were considered to have a poor level of food hygiene and safety | Interview administered questionnaire |
| Lalit et al., 2015 | Food hygiene practice level: respondents who scored less than 80% of their response to the total food hygiene practice-related questions were considered as having poor level of practices. Those who scored equal to or more than 80% were considered as having a good level of practice. | Interview administered questionnaire |
| Shumi et al., 2021 | Food hygiene practice level: respondents who scored less than 70% of their response to the total (21) food hygiene practice-related questions were considered as having poor level of practices. Those who scored equal to or more than 70% were considered as having a good level of practice. | Interview administered questionnaire |
